# Barriers to Hepatitis C Treatment and Interest in Telemedicine-Based Care Among Clients of a Syringe Access Program

**DOI:** 10.1093/ofid/ofae088

**Published:** 2024-02-13

**Authors:** Dorothy E Loy, Kevin Kamis, Ruth Kanatser, Sarah E Rowan

**Affiliations:** Division of Hospital Medicine, Department of Medicine, University of Colorado, Aurora, Colorado, USA; Public Health Institute at Denver Health, Division of HIV/STI/Viral Hepatitis, Denver, Colorado, USA; Harm Reduction Action Center, Denver, Colorado, USA; Public Health Institute at Denver Health, Division of HIV/STI/Viral Hepatitis, Denver, Colorado, USA; Division of Infectious Diseases, Department of Medicine, University of Colorado, Aurora, Colorado, USA

## Abstract

**Background:**

Sharing equipment for injection drug use is the most common mode of hepatitis C virus (HCV) transmission in the United States, yet people who inject drugs (PWID) historically have low rates of HCV treatment. New strategies are needed to expand access to HCV treatment among PWID. Co-locating HCV treatment at syringe access programs (SAPs) reduces barriers to treatment, and telemedicine-based treatment programs could expand access further.

**Methods:**

To evaluate interest in a co-localized or telemedicine-based program at an SAP in Denver, Colorado, we surveyed 171 SAP clients to understand barriers to HCV treatment and comfort with various appointment modalities.

**Results:**

Eighty-nine of the surveyed SAP clients (52%), 50 of whom had not completed treatment, reported current or prior HCV infection. The most commonly cited reasons for not seeking HCV treatment were ongoing drug use, logistic barriers, and medical system barriers. Eighty-eight percent of clients with HCV reported that they would be more likely to get treatment if they were able to do so at the SAP, and the rate was higher among people who reported reluctance to seek medical care in general (98% vs 77%, *P* = .011). In-person appointments were preferred, though 77% of respondents were comfortable with a video appointment. However, only 60% of SAP clients reported having access to a phone, and fewer (48%) had access to video capability.

**Conclusions:**

These findings suggest that telemedicine-based treatment at an SAP could improve access to HCV treatment, but successful implementation would require attention to barriers impacting clients’ ability to participate in telemedicine appointments.

## Teaser

Syringe access program (SAP) clients report high rates of hepatitis C virus (HCV) infection but limited access to treatment. They reported being more likely to get treated for HCV at the SAP and are comfortable with telemedicine-based appointments.

The incidence of hepatitis C virus (HCV) infection has increased in the United States with the worsening of the opioid epidemic [1, 2], with an estimated 69 900 acute hepatitis infections in 2021 [2]. Although sharing equipment for intravenous drug use is the most common mode of HCV transmission in the United States [2], treatment of HCV remains low among people who inject drugs (PWID) [3, 4]. This is despite the recommendation from expert groups that ongoing intravenous drug use is not a contraindication to treatment [5] and evidence that treatment success rates are high in populations of PWID [6–8]. Improvement of HCV treatment rates among PWID would reduce HCV-related morbidity and mortality and decrease transmission [5, 9].

Low HCV treatment rates among PWID have been attributed to several factors. At the individual level, PWID may lack regular access to medical care, face housing instability, have difficulty accessing health insurance [3, 10], or experience competing priorities that limit their ability to complete HCV treatment [3]. At the interpersonal and structural levels, PWID are more likely to face stigma and reluctance to initiate treatment on the part of medical providers [11, 12], and many state Medicaid programs require evidence of sobriety before approving payment for medication to treat HCV infection [13].

There is evidence that removing structural barriers to treatment can improve treatment uptake and patient outcomes among PWID. Several programs have provided HCV treatment at syringe access programs (SAPs), facilities that promote harm reduction strategies among PWID through needle and syringe provision and other services. Studies of these interventions suggest that co-locating harm reduction services and HCV treatment is an effective way to provide HCV care for PWID [14–19].

While co-locating services is ideal in many circumstances, logistical challenges can present obstacles to this approach. Telemedicine-based HCV treatment is an attractive way to connect underserved populations to specialty care. Studies have shown that HCV can be successfully treated using telemedicine-based approaches in many different settings in the United States, including in rural areas, departments of corrections, and substance use disorder treatment clinics [20–22]. During the coronavirus disease 2019 pandemic, an SAP in New Haven, Connecticut transitioned to a partly telemedicine-based treatment model and demonstrated successful HCV treatment outcomes among PWID [23]. A large survey of SAPs conducted in 2020 found that approximately 30% of SAPs nationwide had incorporated some telemedicine-based health services into their center's harm reduction programs, though most did not provide HCV treatment [24]. Telemedicine-based care requires that the patient has access to appropriate technology to participate in appointments and is comfortable with and willing to utilize the services offered.

SAPs in Denver, Colorado currently refer clients to clinic-based HCV treatment providers. Given the data indicating the effectiveness of other treatment models, including both co-localized and telemedicine-based care at SAPs, we sought to assess HCV treatment status, current barriers to HCV-related care, and comfort with and barriers to telehealth models among clients at an SAP. Results of this survey will inform design and implementation of HCV treatment programs targeting PWID who utilize SAPs.

## METHODS

### Setting

The Harm Reduction Action Center (HRAC) is Colorado's largest public health agency that works specifically with PWID. Clients have access to sterile syringes and other harm reduction supplies. HRAC also offers testing services, health education, and referrals for treatment of human immunodeficiency virus (HIV), HCV, and sexually transmitted infections. One referral site for the treatment of HCV is the Public Health Institute at Denver Health (PHIDH), a local public health agency embedded within Denver Health and Hospital Authority, the City and County of Denver's largest safety-net health system. PHIDH houses the Denver Health Infectious Diseases Clinic, one of metro Denver's primary HCV treatment providers.

### Survey Development and Implementation

We developed a 31-question survey in consultation with HRAC staff, HRAC clients, and HCV treatment providers at PHIDH. Survey domains included demographics, HCV testing history and treatment experiences, barriers and preferences for accessing general medical care, and specific details about optimizing HCV treatment success if offered at the SAP. The full survey instrument is available in the Supplementary Materials.

Convenience sampling was used to approach SAP clients for participation in the survey. Participation was limited to once per person. The survey was administered during normal operating hours twice weekly during the month of September 2021. The survey was administered on iPads using the secure, Health Insurance Portability and Accountability Act (HIPAA)–compliant, web-based application REDCap (Research Electronic Data Capture [25]). Clients could fill out the survey on their own or with assistance from a project volunteer. All survey responses were entered anonymously. Survey participants were compensated for their time with a preactivated $15 Visa gift card.

### Data Analysis

We analyzed data from all survey respondents on questions relating to demographics, HCV testing history, and general preferences for medical care. We restricted assessment of treatment history to the 89 SAP clients who self-reported a prior positive HCV test, as defined by choosing one of the following survey responses when asked about most recent HCV test: “Yes, I was positive but I don’t know what kind of test it was,” “Yes, I had a positive hepatitis C antibody test but no virus was detected in my blood,” or “Yes, I had a positive hepatitis C antibody test and virus was detected in my blood.” We then restricted our assessment of barriers to treatment to the subset of 50 of these 89 clients who indicated possible or likely need for treatment, specifically clients with last reported HCV test result of “Yes, I was positive but I don’t know what kind of test it was” and “Yes, I had a positive HCV antibody test and virus was detected in my blood” who also reported treatment history of “No, I haven’t tried to get treatment” or “I tried to get treatment but I was never able to.”

Numerical survey responses were analyzed using Microsoft Excel version 16.16.27. Free text responses were grouped by recurrent themes and categories based on co-author review and consensus. Graphs of responses were generated using RStudio [26] and compiled in Adobe Illustrator 2023. Fisher exact tests were used to compare associations between categorical variables related to HCV treatment history and preferences.

### Patient Consent Statement

The goal of this project was to explore opportunities to improve the coordination of HCV referral and care delivery between HRAC and PHIDH and to inform program design for a possible telehealth HCV treatment program. The project was reviewed by the Quality Improvement Committee of Denver Health, which is authorized by the Colorado Multiple Institutional Review Board at the University of Colorado (COMIRB) and was determined not to be human subjects research. As such, the project did not require further COMIRB review. The project did not include factors necessitating patient consent as no protected health information was collected.

## RESULTS

### Demographics

A total of 171 SAP clients completed surveys, representing approximately 20% of the 797 unique individuals who received services at HRAC that month. The median age of respondents was 37 years (interquartile range, 31–45 years), and most clients were male (82%; Table 1). Most identified as non-Hispanic White (63%), followed by Latino/Latina/Latinx (15%). Unstable housing was commonly reported with 74% of survey respondents experiencing homelessness and only 13% currently living in permanent housing. Ninety-three percent of surveyed HRAC clients reported having health insurance (including Medicaid), though only 31% had a primary care provider (PCP).

**Table 1.** Sociodemographic Data From Surveyed Group

Variable	No. (%)^a^
Age, y	
18–30	36 (21)
31–40	72 (42)
41–50	40 (23)
51–60	17 (10)
≥61	4 (2)
Did not answer	2 (1)
Gender	
Male	140 (82)
Female	27 (16)
Transgender female	4 (2)
Race/ethnicity	
Non-Hispanic White	107 (63)
Latino/a/x (all races)	26 (15)
American Indian/Alaska Native	11 (6)
Mixed race	9 (5)
Non-Hispanic Black	8 (5)
Unknown/other	8 (5)
Native Hawaiian/Pacific Islander	2 (1)
Housing situation in past 3 mo	
Permanent housing	22 (13)
Nonpermanent housing	19 (11)
Homeless	127 (74)
Prefer not to respond	3 (2)
Currently has health insurance	
Yes^b^	159 (93)
No	12 (7)
Has primary care provider	
Yes	53 (31)
No	117 (68)
Did not answer	1 (1)

^a^Total responses vary as responses were not required.

^b^Includes Medicaid.

### HCV Testing Status

**Figure 1.**
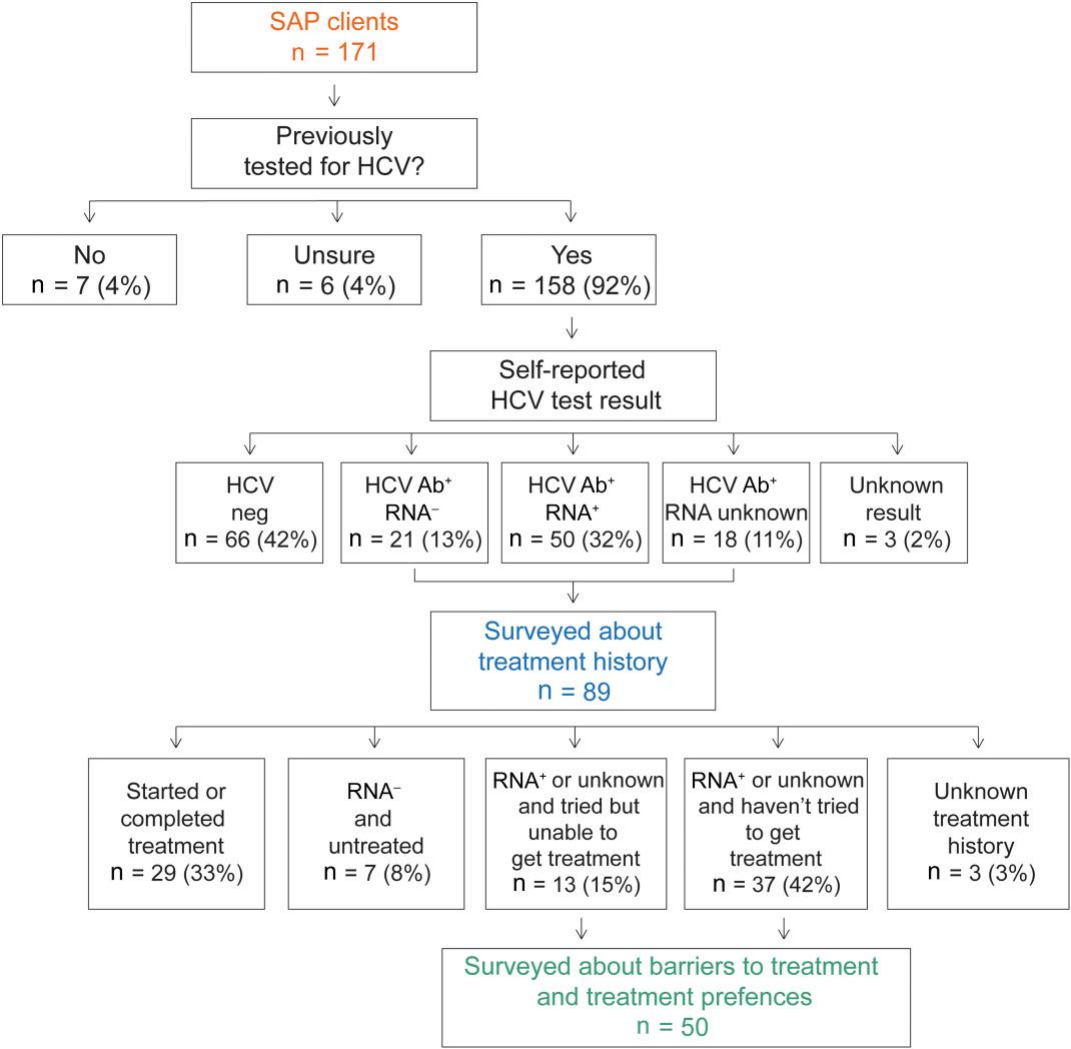
Hepatitis C virus (HCV) testing history and self-reported HCV test results among surveyed syringe access program (SAP) clients. SAP clients were asked about HCV testing status and preferred treatment modalities. Clients who had been tested for HCV indicated their most recent HCV test result; here, “HCV neg” indicates negative antibody test, “HCV Ab^+^ RNA^–^” indicates positive antibody test with negative RNA test, “HCV Ab^+^ RNA^+^” indicates both antibody and RNA test positive, and “HCV Ab^+^ RNA unknown” corresponds to the survey answer “Yes, I was positive but I don't know what kind of test it was.” “Unknown result” groups the survey responses “Yes, I was tested but I do not know what the result was” and “Yes, but I was only tested for hep C antibodies (the initial screening test) and did not have confirmatory testing (RNA test) to see if I had virus in my blood.” The subset of patients who reported a positive HCV antibody test were asked about whether they had gotten treatment, and those reporting no prior treatment and positive or unknown HCV RNA result were asked about treatment preferences.

Among those surveyed, 158 respondents (92% of 171 respondents) reported prior testing for HCV (Figure 1). When asked about most recent test result, 66 of the 158 clients who had been tested reported a negative HCV screening test and 3 did not know the result. Of the 89 clients who reported a positive HCV test, 50 reported test results consistent with current HCV infection (HCV RNA detected) and 18 reported a positive test result but were not sure what type of test it was (which presumably indicates a positive antibody screening test, with unknown RNA status).

### HCV Treatment History

To assess progression along the HCV care continuum, the 89 SAP clients who reported a prior positive HCV test result were asked about their treatment history (Figure 1). Three clients did not respond or were unsure if they had gotten treatment. Twenty-nine clients had started (n = 5) or completed (n = 24) treatment, though 2 clients indicated they were reinfected after treatment. Seven clients reported testing negative for HCV RNA and were not treated. The remaining 50 clients with definite or possible HCV infection (HCV RNA positive or HCV RNA unknown) had never started treatment. Thirteen of these 50 clients had tried to get treatment but couldn’t, and the remaining 37 had not tried to get treatment.

To examine the relationship between having a PCP and being treated for HCV, we excluded the 7 people who were untreated but reported their last RNA test was negative (as they would presumably not need treatment). Of remaining 79 SAP clients with known treatment history and prior positive HCV test, the percentage of people who had completed HCV treatment was higher among those who reported having a PCP: 15 of 24 (63%) clients with a PCP had completed HCV treatment as compared to 14 of 55 (25%) clients without a PCP (*P* = .0024). In addition, clients with permanent housing were also more likely to have been treated for HCV when compared to clients without permanent housing: 6 of 8 (75%) clients with permanent housing had completed HCV treatment versus 23 of 71 (32%) clients without stable housing (*P* = .046).

### Barriers to HCV Treatment

**Figure 2.**
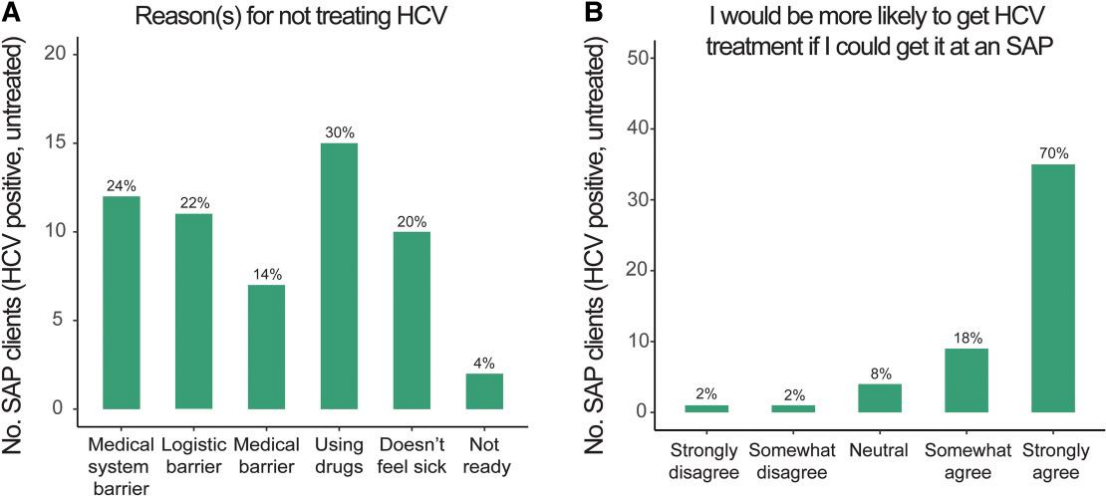
Barriers to hepatitis C virus (HCV) treatment among syringe access program (SAP) clients. *A*, SAP clients who had not completed treatment and who reported that they had a positive HCV antibody test with a positive or unknown RNA test result (n = 50) were asked the reason(s) they had not sought or completed treatment (clients able to select all barriers that applied). *B*, The same clients (n = 50) were then asked if they would be more likely to get treatment for HCV if they could do so at the SAP. For each graph, the percentage of surveyed clients who chose each response is indicated in text over corresponding bar. Bar color corresponds to subset of SAP clients surveyed as described in Figure 1.

We next asked clients with definite or possible HCV infection who had not started treatment about barriers to HCV treatment (n = 50; Figure 1). Clients could select all the barriers to treatment that applied to them or use a free text response. Most clients selected a single barrier, but 7 clients selected >1. Survey and free text responses were grouped into broad categories as shown in Supplementary Table 1. Many clients (n = 12) reported a medical system barrier that had prevented them from getting treatment, most commonly never having been offered HCV treatment by their medical provider and not knowing about treatment options (Figure 2*A* and Supplementary Table 1). Other medical system barriers noted included lack of insurance, lack of treatment in correctional facilities, and being told by a provider that they were not eligible for treatment due to substance use. A similar number of clients (n = 11) cited logistic constraints such as not having time for frequent appointments and difficulty navigating the process of getting treatment. Fifteen clients reported that they considered their ongoing drug use to be a barrier to HCV treatment, and 11 clients said lack of symptoms prevented them from seeking treatment. Seven people referenced perceived medical barriers, such as concern about side effects of treatment, impact on HIV treatment, or medical comorbidities that make treatment challenging. Only 2 clients said they were not ready to seek treatment. Of note, 4 people used the free text option to indicate that they no longer needed treatment (eg, responded “hep c went away on its own” or “body treated itself”; Supplementary Table 1) despite earlier reporting a positive or unknown HCV RNA result.

We also asked about reluctance to seek medical care in general, outside of HCV-specific concerns. More than half of clients surveyed (90 of 171 clients [53%]) reported reluctance to be seen for a medical problem at a doctor's office or clinic. In free text responses (Supplementary Table 2), the most common reasons cited were concerns about stigma and discrimination, fear or anxiety about bad results, and a desire to avoid bad news (Table 2). Additional reasons included financial or time constraints and legal concerns.

**Table 2.** Reasons for Reluctance to Seek Medical Care

Category^a^	No. (%^b^)
Stigma or discrimination	17 (19)
Fear of or anxiety with medical establishment	16 (18)
Negative opinion of medical establishment	14 (16)
Physical or time constraints	12 (13)
Fear or avoidance of bad results	9 (10)
Prior negative experience	8 (9)
Embarrassment	5 (6)
Financial	4 (4)
Available options don’t meet needs	4 (4)
Legal concerns	4 (4)
Impact on substance use	3 (3)
Not a priority	3 (3)
Privacy concerns	1 (1)

^a^For original text of each response, see Supplementary Table 2.

^b^Some clients cited multiple reasons; the percentage is derived using total number of clients surveyed (n = 89) as denominator.

We asked respondents with definite or possible current HCV infection who had not started treatment (n = 50) whether they would be more likely to get treated for HCV if they were able to do so at the SAP. Eighty-eight percent (n = 44) of these individuals either agreed or strongly agreed that they would be more likely to get treated for HCV if they could do so at the SAP (Figure 2*B*).

### Treatment Modality Preferences Among SAP Clients

**Figure 3.**
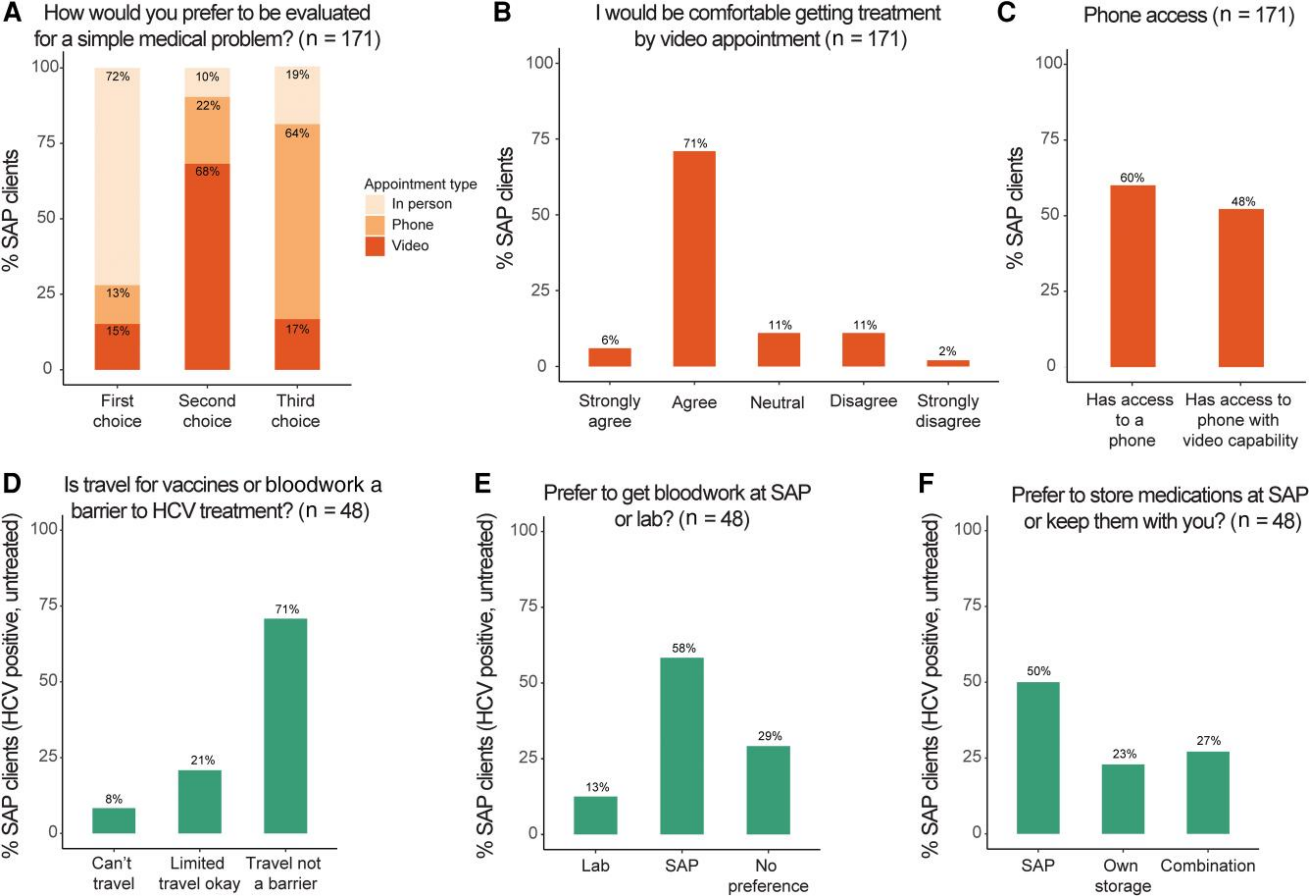
Comfort with and barriers to telemedicine-based appointments and hepatitis C virus (HCV) treatment at a syringe access program (SAP). *A*, All surveyed SAP clients, regardless of HCV test status (N = 171), were asked “How would you prefer to be evaluated for a simple medical problem? Please rank your first, second, and third choice.” *B*, They were next asked to rate how strongly they agreed or disagreed with the statement “I would be comfortable getting treatment by video appointment with a doctor.” *C*, All clients were asked whether they usually have access to a phone and if the phone has Facetime or other video capability. *D–F*, Forty-eight of 50 SAP clients with definite or likely HCV infection and no prior treatment answered the following questions: if having to travel for bloodwork or vaccines would prevent them from getting treatment at the SAP (*D*), whether they preferred to get bloodwork done at the SAP by appointment at a laboratory or on their own time (*E*), and whether they would prefer to store medications at the SAP or to keep medications with them (*F*). For each graph, the percentage of surveyed clients is plotted and indicated in text over corresponding bar. Bar color corresponds to subset of SAP clients surveyed as described in Figure 1.

We asked all clients surveyed to rank their preferred medical care setting, choosing between in-person appointments, video appointments, and phone appointments. The majority ranked in-person appointments as their first choice of treatment modality (72%; Figure 3*A*). Phone and video appointments were the first choice of 13% and 15% of survey respondents, respectively. Sixty-eight percent of respondents selected a video appointment as their second choice (Figure 3*A*). Despite not being most clients’ first choice of treatment modality, 77% of respondents either agree or strongly agree that they would be comfortable with a video appointment (Figure 3*B*). However, only 60% reported access to a phone and only 48% had access to a phone with video capability (Figure 3*C*).

### Bloodwork Location and Medication Storage Preferences

HCV treatment requires blood tests and taking a daily medication for 8–12 weeks. We restricted questions about ability to access these services to the group of individuals with definite or possible HCV infection who had not started treatment (n = 50). Forty-eight of these 50 clients answered the questions. Most (71%) reported that travel to a third-party location for laboratory work and/or vaccination would not be a barrier for them (Figure 3*D*), though the majority (58%) would prefer to get laboratory work done at the SAP by appointment (Figure 3*E*). A high percentage of clients indicated that they would prefer to store medication on-site at the SAP some (27%) or all (50%) of the time (Figure 3*F*).

### Reluctance to Seek Medical Care Is Associated With Preference for HCV Treatment at the SAP

Self-reported reluctance to seek medical care was associated with agreeing or strongly agreeing that the client would be more likely to get treated for HCV on-site at the SAP. Sixty-eight of the 89 clients who reported a prior positive HCV antibody test result answered questions about HCV treatment at the SAP and their reluctance to seek medical care. Forty-one of 42 clients (98%) who were reluctant to seek medical care agreed they would prefer treatment at the SAP, versus 20 of 26 (77%) of clients who said they had not been reluctant to get medical care in the past (*P* = .011). There was no association between having PCP or stable housing and preference for treatment at the SAP.

## DISCUSSION

Better understanding of the barriers to HCV treatment among PWID and strategies to improve treatment rates are essential in combating the rising rate of HCV incidence in the United States [9]. In this report, we present results from a survey of 171 SAP clients that identify many of the current barriers to HCV treatment in this population and assess comfort with and barriers to in-person and telemedicine-based appointments.

Most SAP clients reported prior HCV testing and knowledge of their HCV status. However, only half of the individuals who reported HCV testing results consistent with definite or possible HCV infection had tried to access treatment. A low rate of HCV treatment initiation among PWID is consistent with other studies [27–29], and indicates that the transition from diagnosis to treatment represents an important intervention point. In our surveyed population, the majority of people who had not received treatment cited either a logistic barrier or a medical system barrier. One medical system barrier cited was not being offered treatment after a positive test, which has previously been noted to be a problem among PWID [30]. In our population, 20% of people with HCV cited lack of symptoms as a reason for not seeking treatment, similar to a recent survey of PWID in California [31] but slightly less emphasized in this group relative to others [27, 32]. Interestingly, concerns about side effects were very rarely cited in this population in contrast to other studies [3, 30, 31], suggesting that people may be aware that modern treatments have fewer side effects than older, interferon-based therapies. It is clear that the reasons for not seeking HCV treatment are complex and multifactorial among PWID, but our data suggest that many SAP clients would pursue treatment for HCV if logistic and system issues can be overcome.

Although current drug use is not a medical contraindication to HCV treatment [5], and most state Medicaid programs (including Colorado) have removed sobriety requirements for treatment [13], 30% of our survey respondents indicated that ongoing drug use was a reason they had not been treated. Indeed, 1 client in this survey cited the primary reason for not seeking treatment being “told he had to be clean” and several said that treatment had not been offered by their doctor. This is consistent with studies among PWID in geographically diverse settings [31, 33–35], and likely reflects widespread persistence of outdated ideas that HCV treatment should be reserved for people whose drug use is in remission. However, it is important to acknowledge that ongoing drug use is a complex issue, representing both a reason a person could be denied treatment by a physician or insurance company and a personal barrier to treatment of HCV. Specifically, other surveys and structured interviews of PWID found ongoing drug use was a competing priority for time and resources [33, 35] and was associated with a feeling of futility for some people who are worried about reinfection [32, 33]. Indeed, 1 client in our survey said they were reluctant to get HCV treatment “because I still live the lifestyle that got me hep C, so don’t want to get treated and just contract it again.” At the same time, Tsui et al found that preventing spread to others was a motivating factor for treatment among PWID [33]. Co-localizing HCV treatment at an SAP could help combat the narrative that sobriety is a prerequisite to HCV treatment, and could also represent an opportunity to frame HCV treatment as a form of harm reduction.

Many SAP clients we surveyed expressed reluctance to seek medical care in traditional settings due to concerns about stigma and discrimination, which is a common theme noted in other studies on this subject [31, 33–36]. Clients in our survey shared experiences where they were “treated harshly and judged by doctors” and reported being “afraid they'll find marks on my arm, judge me for using heroin.” As harm reduction centers such as SAPs tend to elicit feelings of confidence and acceptance rather than fear or concern about stigmatization [34, 36], treatment of HCV within an SAP is a promising approach to mitigate this barrier. Indeed, several studies have shown that offering HCV treatment to people at an SAP [14–19, 23, 37, 38] or an opioid treatment program (OTP) [20] can result in high rates of cure. Furthermore, this community-based approach to HCV treatment has been shown to be acceptable or even preferred among PWID [35, 39–41]. In addition to mitigating stigma, on-site treatment in an SAP also decreases access barriers and increases the perceived urgency of treatment [35], which could help increase treatment rates, as indicated by the large number of clients in our survey who cited lack of symptoms as the main barrier for not seeking treatment for HCV.

In an effort to expand access to HCV treatment while mitigating the logistic hurdles to opening and staffing a new clinic co-localized in an SAP or OTP, several groups have trialed HCV treatment interventions that are completely or partially telemedicine-based at SAPs [23], OTPs [20], and other addiction treatment centers [42]. A follow-up study of clients who had received telemedicine-based HCV treatment at an OTP showed that people found it very convenient to get treatment at a location they were already visiting on a regular basis [43]. We were interested in which treatment modality—telemedicine versus in-person appointments—would be preferred among clients of an SAP. While most (72%) clients in our survey population preferred in-person appointments, 77% said they would be comfortable with a video appointment. Importantly, we also identified obstacles to providing telemedicine-based HCV care in the surveyed population. First, less than half of individuals surveyed had access to a phone with video capability, a barrier that can be overcome by providing a secure, confidential computer on-site to facilitate telemedicine-based care [20]. Second, most clients were experiencing housing insecurity and would like the option to store medications at the SAP. Finally, most but not all SAP clients would be able to travel for blood tests if needed. These data suggest that a holistic approach offering on-site laboratory services, medication storage, and access to a video-capable computer in a confidential space at the SAP would be critical for success if a purely telemedicine-based approach is implemented.

Our survey data have a few limitations. First, the survey methodology was based on convenience sampling, which could limit generalizability of the results. However, by offering the survey twice weekly over a 1-month period, we were able to enroll a significant number of clients, which likely mitigated this limitation. Second, people with HCV or those interested in HCV treatment might be overrepresented in the study given their voluntary participation in a survey about HCV. Third, grouping of themes that emerged from survey responses was subjective and not based on a preexisting framework. We mitigated the necessarily subjective nature of this grouping by reviewing categories with multiple authors. Finally, all results are self-reported and subject to reliance on the person's memory of their medical history and other recall bias. This includes potential unreliability of self-report of hepatitis C testing and treatment status, which has been noted in other surveys of PWID [31, 41]. Indeed, in a couple of cases the free text responses contradicted self-reported HCV status (as in the case of the client who said their “body treated itself” but also reported that their last RNA test had been positive).

The results of this survey of SAP clients indicate that providing care in a less stigmatizing, more familiar and trusted environment could improve HCV treatment rates among PWID by decreasing logistic and medical system barriers and combating the misconception that sobriety is a prerequisite for treatment. There appears to be enthusiasm for such a program among SAP clients as well as a high degree of preexisting comfort with telemedicine-based care. This suggests that telemedicine-based HCV treatment could be adopted by the population of PWID who use SAPs, though attention should be focused on details of the programmatic design to meet the needs of this historically underserved population.

## Supplementary Material

ofae088_Supplementary_Data

## Supplementary Data

Supplementary materials are available at *Open Forum Infectious Diseases* online. Consisting of data provided by the authors to benefit the reader, the posted materials are not copyedited and are the sole responsibility of the authors, so questions or comments should be addressed to the corresponding author.

## Notes

***Acknowledgments***. We would like to thank Kendra Newman, Kari Duff, and Jesús Chávez-van de Hey for their help with survey collection, and Harm Reduction Action Center clients and staff for their participation and support.

***Author contributions*.** All authors designed the survey. K. K. and S. E. R. administered the survey. D. E. L., K. K., and S. E. R. analyzed the data. D. E. L. wrote the first draft of the manuscript and created figures and tables. All authors edited the manuscript.
