# Supplementary material for: Barriers to Hepatitis C Treatment and Interest in Telemedicine-Based Care Among Clients of a Syringe Access Program
Source: Open Forum Infect Dis. 2024 Feb 13;11(3):ofae088. doi: 10.1093/ofid/ofae088 (PMC10921388; doi:10.1093/ofid/ofae088)
Supplement: ofae088_Supplementary_Data [file ofae088_supplementary_data.zip › SupplementalTable1.pdf]

Response to "If you have hepatitis C but have not sought treatment for it,what was the reason? Please select all that apply."	Number of clients who cited reason	Grouped category
I didn't feel sick	8	Doesn't feel sick
I didn't know about it	2	Medical system barrier
My healthcare provider never offered it	5	Medical system barrier
Treatment was not offered in my primary care clinic	1	Medical system barrier
I didn't have the health insurance or medication coverage to pay for it	1	Medical system barrier
The clinic that offered it was too far from my home	0	Logistic barrier
I didn't have time to attend regular appointments	4	Logistic barrier
I was concerned about the side effects	3	Medical barrier
I am waiting until new therapies become avaliable	0	Medical barrier
I was concerned hepatitis C treatment would negatively impact my HIV treatment	1	Medical barrier
I want to work on other things first / I don't feel ready	1	Not ready
I still use drugs	9	Using drugs
Other; free text responses listed below	25	
Told he had to be clean	1	Using drugs
Because I still live the life style that got me hep c so don't want to get treated and just contract it again	1	Using drugs
Finding stability to take care of all of it	1	Logistic barrier
Haven't had time	1	Logistic barrier
I also have hep b	1	Medical barrier
I was in a 90 day rehab when I was tested, the nurse there told me that I had toseek treatment after leavIng.	1	Medical system barrier
In DOC, didn't have time to finish Tx	1	Medical system barrier
Issues with jail , haven't gotten past the stage of wanting to get tx, miss appointments	1	Logistic barrier
It doesn't seem like a major problem right now	1	Doesn't feel sick
It's not bothering me	1	Doesn't feel sick
Laziness, dope heals me	1 for each barrier	Logistic barrier, Using drugs
Logistic	1	Logistic barrier
Mental disabilities, hard to keep track of dates	1	Medical barrier
Moved from different state	1	Logistic barrier
Process was difficult	1	Logistic barrier
Procrastinate, I'm a mental health pt and I don't get things done that I need to get done	1	Medical barrier
Still using drugs	1	Using drugs
Stopped methodone, couldn't get tx for hep c	1	Using drugs
Waiting on housing. Lord told me not to yet, it's a performance of miracles.	1	Not ready
Wants to be stable on methadone alone	1	Using drugs
Was told it wasn't bad enough to treat yet by provider	1	Medical system barrier
Ab + only currently seeking to start treatment	n/a - no need for treatment, no barrier	No need for treatment
Was tested, never got results back	n/a - no need for treatment, no barrier	No need for treatment
Body treated itself	n/a - no need for treatment, no barrier	No need for treatment
Hep c went away on its own	n/a - no need for treatment, no barrier	No need for treatment
