# Supplementary material for: Barriers to Hepatitis C Treatment and Interest in Telemedicine-Based Care Among Clients of a Syringe Access Program
Source: Open Forum Infect Dis. 2024 Feb 13;11(3):ofae088. doi: 10.1093/ofid/ofae088 (PMC10921388; doi:10.1093/ofid/ofae088)
Supplement: ofae088_Supplementary_Data [file ofae088_supplementary_data.zip › SupplementalTable2.pdf]

Reason for "yes" response to "Have you ever been reluctant to get seen for a medical problem at a doctor's office or clinic?"	Physical or time constraints	Fear or anxiety	Fear or avoidance of bad results	Embarrass-ment	Negative opinon of medical establishments	Financial	Stigma or discrimination	Available options don't meed needs	Prior negative experience	Legal concerns	Privacy	Impact on substance use	Not a priority
**Total**	**12**	**17**	**9**	**5**	**11**	**4**	**18**	**4**	**8**	**4**	**1**	**3**	**3**
"Denver health scares the living shit out of me"; they don't care about if patients get better		1											
Abscess on arm before , freaked me out.									1				
Abscesses - have been treated poorly by staff									1				
Afraid of not being able to get heroin												1	
Afraid of results			1										
Afraid that I will be embarrassed , keep missing appointments, get discouraged				1									
Afraid they would warrant check and arrest them at hospital; afraid of getting sick at hospital										1			
All the time. Afraid they'll find marks on my arm, judge me for using heroin. Afraid I'll lose visitation my kid (e.g. reported to CPS by medical team)							1			1			
Anxiety		1											
Anxiety		1											
Anxiety , panic paranoia		1											
Anxiety, scared about not being able to get what you want		1											
Bc of the views of addiction, don't want to deal with treatment if going to get judged for other things							1						
Because anytime I see one, it's not good. You're always broken somewhere, even mental			1										
Because I was scared of the results			1										
Because I'm afraid of the results or I feel like they'll do nothing.		1	1										
Because of treatment by staff for addiction									1				
Couldn't afford it						1							
Couldn't afford medication and didn't have primary care doctor						1		1					
Deep down I would rather not know what's going on			1										
Didn't want to get arrested, afraid of warrant checking										1			
Didn't want to get HIV testing because already positive. Didn't feel like getting treatment because of the amount of time it would take	1												
Didn't want to go in, some of the nurses can be rough. If they find you're a drug addict they'll kind of tough.							1		1				
Discrimination							1						
Discrimination Based on my Gender and being homeless							1						
Don't have time. Condescending doctors.	1				1								
Don't like doctors					1								
Don't like going to the doctor					1								
Don't like hospitals					1								
Don't like them					1								
Don't trust medical professionals, had really bad er experiences-really stigmatizing					1		1						
Don't want people to know their health information/were afraid of privacy violations											1		
Drug addicts aren't treated the same as people who don't use drugs							1						
Drugs												1	
Embarrassed				1									
Embarrassing to go through				1									
Embarrassment				1									
Fear of discrimination							1						
Fear of doctors and hospitals; remembers when the time when you only went to hospital if you were going to die		1											
Fear of hospitals		1											
Fear of how doctors would treat them because of drug use							1						
Fear of the severity			1										
Felt that the facilities available were unappealing								1					
Financial issues						1							
For me, ignoring a problem until it goes away is what has worked for me, so that's what I do.													1
Getting nervous		1											
Got jumped									1				
Hard to get to	1												
Hate going to doctors, matter of just going					1								
Hate hospitals, especially things w abscess bc of how they look at drug addicts							1						
Hates hospital, tries to do as much as she can to treat myself. When do go in, usually serious (endocarditis)					1								
I hate to be treated like a piece of shit							1						
I have been reluctant due to the fact that I am a drug user.							1						
I just really hate gong to the doctor or hospital					1								
Just don't like hospitals					1								
Just don't want to													1
Lack of insurance at the time						1							
Living situation	1												
Logistics, discrimination	1						1						
Nervous		1											
Non believer					1								
Not enough dope												1	
Not sure if people like me get adequate care							1						
People judge me							1						
Phobia , paranoid		1											
Pinky toe too much pain	1												
Prescribed something he was allergic to									1				
Scared		1											
Scared , embarrassed		1		1									
Scared of doctors		1											
Scared of results			1										
Scared of the outcome			1										
Scared of the results			1										
Scared to get treatment. Scared to have surgery done		1											
Scared, fear		1											
Seems like a waste of time unless it's an emergency health issue	1												
Sometimes I have a fewer that I may have caught something or that it will take too long.													
Stigma. Previously prejudiced doctors							1		1				
The wait , not enough workers	1												
Time consuming and I don't like crowds in waiting rooms	1							1					
Too much to deal with													1
Too weak, blisters	1												
Treat me differently							1						
Treated harshly and judged by doctors because of drug use. Drug use affects the medications prescribed.							1		1				
Warrants, scared they would do warrant checks										1			
Weekend	1												
White coat syndrome', gets anxious going into clinic or hospital		1											
Yes because I'm not able too get to further scheduled appointments.	1												
You never know if you'll get good treatment or not. Have had lots of different answers for same medical issues								1					
