# Supplementary material for: Barriers to Hepatitis C Treatment and Interest in Telemedicine-Based Care Among Clients of a Syringe Access Program
Source: Open Forum Infect Dis. 2024 Feb 13;11(3):ofae088. doi: 10.1093/ofid/ofae088 (PMC10921388; doi:10.1093/ofid/ofae088)
Supplement: ofae088_Supplementary_Data [file ofae088_supplementary_data.zip › HRAC telemed survey CODEBOOK v14SEP2021.pdf]

Codebook

Data Dictionary Codebook

09/14/2021 2:57pm

Collapse all instruments

#	Variable / Field Name	Field Label<br>*Field Note*	Field Attributes (Field Type, Validation, Choices, Calculations, etc.)
Instrument: **HRAC Client Survey: HCV Telemed** (hrac_client_survey_hcv_telemed)		Enabled as survey	Collapse
1	record_id	Record ID	text
2	age	What is your age?	text (integer, Min: 18, Max: 99)
3	gender	What is your gender?	radio<br>1 Male<br>2 Female<br>3 Transgender male<br>4 Transgender female<br>5 Nonbinary<br>6 Gender nonconforming<br>7 Other<br>Custom alignment: LV
4	race	What is your race/ ethnicity? Choose all that apply.	checkbox<br>1 race___1 Native American/American Indian or Alaskan Native<br>2 race___2 Asian<br>3 race___3 Black or African American<br>4 race___4 Hispanic or Latino<br>5 race___5 Native Hawaiian or Other Pacific Island<br>6 race___6 White<br>7 race___7 Other<br>8 race___8 I prefer not to answer<br>Custom alignment: LV
5	housing	Which option best described your housing situation during the past 3 months?	radio<br>1 Permanent housing<br>2 Non-permanent housing (for example, staying with friends)<br>3 Homeless<br>4 I don't know<br>5 I prefer not to answer<br>Custom alignment: LV
6	hrac	How often do you come here to the Harm Reduction Action Center?	radio<br>1 More than once a week<br>2 Once a week<br>3 Once every few weeks<br>4 Once a month or less<br>Custom alignment: LV

7	hcvtest	Have you ever been tested for hepatitis C? If yes, what was the result of the most recent test?	radio<br>1 Yes, I was positive but I don't know what kind of test it was<br>2 Yes, I had a positive hepatitis C antibody test but no virus was detected in my blood<br>3 Yes, I had a positive hepatitis C antibody test and virus was detected in my blood<br>4 Yes, my hepatitis C testing was completely negative<br>7 Yes, I was tested but I do not know what the result was<br>5 I have never been tested for hepatitis C<br>6 I don't know/I am unsure<br><br>Custom alignment: LV
8	treatment<br>Show the field ONLY if:<br>[hcvtest]<>'5'	Have you ever tried to get treatment for hepatitis C?	radio<br>1 Yes, I was treated and cured<br>7 Yes, I was treated and cured, but then I got hep C again<br>8 Yes, I was treated but I did not get cured<br>2 I was treated but I'm not sure if I was cured<br>3 I started treatment but wasn't able to finish it<br>6 I tried to get treatment but I was never able to<br>4 No, I haven't tried to get treatment<br>5 I have never been diagnosed with hepatitis C<br><br>Custom alignment: LV

9	reasonsfornottesting<br>Show the field ONLY if:<br>[hcvtest] = '1' or [hcvtest] = '3'	If you have hepatitis C but have not sought treatment for it, what was the reason? Please select all that apply.	checkbox<br>1 reasonsfornottesting___1 I didn't know about it<br>2 reasonsfornottesting___2 My healthcare provider never offered it<br>3 reasonsfornottesting___3 Treatment was not offered in my primary care clinic<br>4 reasonsfornottesting___4 I didn't have the health insurance or medication coverage to pay for it<br>5 reasonsfornottesting___5 I was concerned about the side effects<br>6 reasonsfornottesting___6 I am waiting until new therapies become available<br>7 reasonsfornottesting___7 The clinic that offered it was too far from my home<br>8 reasonsfornottesting___8 I was concerned hepatitis C treatment would negatively impact my HIV treatment<br>9 reasonsfornottesting___9 I didn't feel sick<br>10 reasonsfornottesting___10 I didn't have time to attend regular appointments<br>12 reasonsfornottesting___12 I still use drugs<br>13 reasonsfornottesting___13 I want to work on other things first / I don't feel ready<br>11 reasonsfornottesting___11 Other<br>Custom alignment: LV
10	treatmentother<br>Show the field ONLY if:<br>[reasonsfornottesting(11)] = '1'	If other, please specify	notes<br>Custom alignment: LV
11	lasttest<br>Show the field ONLY if:<br>[hcvtest] <> '5'	When were you last tested for hepatitis C?	radio<br>1 Less than 1 month<br>2 1-6 months ago<br>3 7-12 months ago<br>4 More than a year ago<br>5 I don't know/ I'm not sure<br>Custom alignment: LV
12	testingathrac<br>Show the field ONLY if:<br>[hcvtest] <> '5'	Have you been tested for hepatitis C at the Harm Reduction Action Center (HRAC)?	radio<br>1 Yes<br>2 No<br>3 I don't remember<br>Custom alignment: LV

13	testfrequency<br>Show the field ONLY if:<br>[hcvtest] <> '5'	How frequently do you get tested for hepatitis C?	radio<br>1 Less than once per year<br>2 About once per year<br>3 About 2-3 times per year<br>4 Once a month or more<br>5 I don't know/ I'm not sure<br>Custom alignment: LV
14	travel	Have you ever been unable to get treatment for a medical problem because you didn't have transportation?	radio<br>1 Yes<br>2 No<br>Custom alignment: LV
15	reluctantfortreatment	Have you ever been reluctant to get seen for a medical problem at a doctor's office or clinic?	radio<br>1 Yes<br>2 No<br>Custom alignment: LV
16	yesreluctanttotesting<br>Show the field ONLY if:<br>[reluctantfortreatment] = '1'	If yes, why?	notes<br>Custom alignment: LV
17	videoappt	Have you ever met with a doctor or other medical provider through a video appointment?	radio<br>1 Yes<br>2 No<br>Custom alignment: LV
18	telephoneappt	Have you ever met with a doctor or other medical provider through a telephone appointment?	radio<br>1 Yes<br>2 No<br>Custom alignment: LV
19	comfortable	I am comfortable getting treatment for a medical problem in person at a doctor's office.	radio<br>1 Strongly disagree<br>2 Disagree<br>3 Neither agree nor disagree<br>4 Agree<br>5 Strongly agree<br>Custom alignment: LV<br>Field Annotation: @HIDECHOICE='1,5'
20	videoappointment	I would be comfortable getting treatment for a medical problem over a video appointment with a doctor.	radio<br>1 Strongly disagree<br>2 Disagree<br>3 Neither agree nor disagree<br>4 Agree<br>5 Strongly agree<br>Custom alignment: LV<br>Field Annotation: @HIDECHOICE='1,5'

21	telephoneappt1	I would be comfortable getting treatment for a medical problem over a telephone appointment with a doctor.	radio<br>1 Strongly disagree<br>2 Disagree<br>3 Neither agree nor disagree<br>4 Agree<br>5 Strongly agree<br><br>Custom alignment: LV<br>Field Annotation: @HIDECHOICE='1,5'
22	comfortable_at_droffice	Section Header: *How would you prefer to be evaluated by a medical provider for a simple medical problem? Please rank your first, second, and third choice.*<br>in person at a doctor's office	radio (Matrix - ranking)<br>1 First Choice<br>2 Second Choice<br>3 Third Choice
23	comfortable_over_video	video appointment with a doctor	radio (Matrix - ranking)<br>1 First Choice<br>2 Second Choice<br>3 Third Choice
24	comfortable_over_telephone	telephone appointment with a doctor	radio (Matrix - ranking)<br>1 First Choice<br>2 Second Choice<br>3 Third Choice
25	testinghrac<br>Show the field ONLY if:<br>[hcvtest] = '1' or [hcvtest] = '3'	I would be more likely to get treated for hepatitis C if I was able to do it at the Harm Reduction Action Center.	radio<br>1 Strongly disagree<br>2 Disagree<br>3 Neither agree nor disagree<br>4 Agree<br>5 Strongly agree<br><br>Custom alignment: LV
26	dailymeds<br>Show the field ONLY if:<br>[hcvtest] = '1' or [hcvtest] = '3'	Hepatitis C treatment requires taking a medication daily for 8-12 weeks. If you were being treated for hepatitis C at the Harm Reduction Action Center, would it be helpful for your medications to be stored at Harm Reduction Action Center or would you prefer to keep them with you?	radio<br>1 Store them at Harm Reduction Action Center<br>2 Keep with me<br>3 Combination of both as needed<br><br>Custom alignment: LV
27	blooddraws<br>Show the field ONLY if:<br>[hcvtest] = '1' or [hcvtest] = '3'	If you were being treated for hepatitis C at the Harm Reduction Action Center, would having to travel to Denver Health for vaccines or blood draws prevent you from completing hepatitis C treatment?	radio<br>1 Yes, if I had to go more than a couple of times it would be a problem<br>2 Yes, even if I had to go once it would be a problem<br>3 No<br><br>Custom alignment: LV
28	blooddrawlocation<br>Show the field ONLY if:<br>[hcvtest] = '1' or [hcvtest] = '3'	Would you prefer to go to the doctor's office lab to get blood tests on your own schedule or get blood tests at Harm Reduction Action Center by appointment?	radio<br>1 Lab on my own time<br>2 Harm Reduction Action Center by appointment<br>3 No preference<br><br>Custom alignment: LV

29	healthinsurance	Do you have health insurance or health care coverage, including Medicaid?	radio<br>1 Yes<br>2 No<br><br>Custom alignment: LV
30	pcp	Do you have a primary care doctor or primary care provider?	radio<br>1 Yes<br>2 No<br><br>Custom alignment: LV
31	accesstophone	Do you usually have access to a phone? (Not including the phone at HRAC)	radio<br>1 Yes<br>2 No<br><br>Custom alignment: LV
32	accesstofacetime<br>Show the field ONLY if:<br>[accesstophone] = '1'	Does your phone have Facetime or another type of option for video-conferencing?	radio<br>1 Yes<br>2 No<br>3 I don't know<br><br>Custom alignment: LV
33	importanthealthissues	What health issues are the most important to you at this time?	notes<br>Custom alignment: LV
34	notes	Please enter any additional notes related to this survey	notes
35	staffmemberhelp	Did a Harm Reduction Action Center or Denver Health staff member help you answer this questionnaire?	radio<br>Yes I had help<br>No I filled in my responses by myself<br><br>Custom alignment: LV
36	hrac_client_survey_hcv_telemed_complete	Section Header: *Form Status*<br>Complete?	dropdown<br>0 Incomplete<br>1 Unverified<br>2 Complete
